# Precision Nutrition: The Hype Is Exceeding the Science and Evidentiary Standards Needed to Inform Public Health Recommendations for Prevention of Chronic Disease

**DOI:** 10.1146/annurev-nutr-061021-025153

**Published:** 2023-08-21

**Authors:** Regan L. Bailey, Patrick J. Stover

**Affiliations:** Institute for Advancing Health through Agriculture and Department of Nutrition Science, Texas A&M University, College Station, Texas, USA

**Keywords:** precision nutrition, nutrient requirements, nutrition policy, public health, biological variation, dietary recommendations

## Abstract

As dietary guidance for populations shifts from preventing deficiency disorders to chronic disease risk reduction, the biology supporting such guidance becomes more complex due to the multifactorial risk profile of disease and inherent population heterogeneity in the diet–disease relationship. Diet is a primary driver of chronic disease risk, and population-based guidance should account for individual responses. Cascading effects on evidentiary standards for population-based guidance are not straightforward. Precision remains a consideration for dietary guidance to prevent deficiency through the identification of population subgroups with unique nutritional needs. Reducing chronic disease through diet requires greater precision in (*a*) establishing essential nutrient needs throughout the life cycle in both health and disease; (*b*) considering effects of nutrients and other food substances on metabolic, immunological, inflammatory, and other physiological responses supporting healthy aging; and (*c*) considering healthy eating behaviors. Herein we provide a template for guiding population-based eating recommendations for reducing chronic diseases in heterogenous populations.

## 1. INTRODUCTION

Fundamental advances in nutrition science and their translation to improve public health evolve continuously. The field of nutrition has progressed from early discoveries of essential micronutrients to quantifying requirements for preventing deficiency disorders to ensuring adequate intakes through recommendations and fortification practices and, most recently, to a holistic focus on dietary patterns that reduce chronic disease risk. Diet is among the most promising modifiable factors to promote human health, creating an urgency to develop scientifically grounded and evidence-based public health strategies that reduce high rates of nutrition-related chronic diseases. Governments and related entities provide broad-based dietary and nutrition guidance with a goal of improving population health through dietary recommendations. Nutrient- and food-based recommendations encourage consumption of specific nutrients, food components, foods, and dietary patterns at specified intake levels; this reflects a broad-based public health nutrition approach and has been effective in addressing diseases of nutritional deficiencies (117). In general, existing authoritative nutritional guidance has taken this population-based approach, under the assumption that all individuals in the population or within a limited number of population subgroups respond similarly to food and nutrient exposures. This approach has successfully addressed deficiency disorders of specific nutrients but the approach is more complicated when turning toward the role of nutrition in chronic diseases.

The past three decades have seen unprecedented increases in the incidence of diet-related chronic diseases and their associated impact on health-care costs, motivating efforts to extend the goal or end point of nutrient- and food-based guidance and policies to include chronic disease reduction (117). The multifactorial etiology of chronic diseases, the complexity of food composition and the multitude of interactions of foods with physiological systems, nutrition behaviors, the aging process, and knowledge of human biological variation among individuals all contribute to differences in the diet–disease relationship, indicating the need for greater precision in achieving health through diet that must be largely reflected in more nuanced dietary guidance, practice, and food policy.

This review summarizes the biological premise as well as challenges and opportunities in achieving the aspirational goal of deriving food- and nutrient-based guidance for chronic disease risk reduction through precision nutrition. Precision nutrition is based on the concept that population subgroups, rather than the individual or the entire population, may react in similar ways to dietary exposures (i.e., similar host responses) and therefore understanding this variation in response enables our ability to tailor recommendations that are more specific than those given at the population level but that are more broad based than personalized recommendations (Table 1); these definitions put forth are not the first to try to describe the nuances of these terms, and our proposed list builds off the original work of others (14, 57, 62, 89). This work describes the historical progression of nutrition guidance, and it provides background on the biological and contextual factors that contribute to variability in the human response to diet. We propose some terms to advance the future of precision nutrition decision-making, realizing that we are not the first in the field to do so. This work concludes with knowledge gaps and other gaps that must be considered in the future as to what precision nutrition is and how its potential can be utilized or maximized.

## 2. THE BIOLOGICAL BASIS OF FOOD AND NUTRIENT REQUIREMENTS

Throughout human history, regional food landscapes have shaped the human genome for survival and populational expansion within a multitude of local environmental contexts. These historical adaptations now contribute to modern-day variations in risk for chronic disease incidence in the context of a changing and increasingly maladaptive food ecosystem for many individuals (115). Food availability and food composition have been among the primary environmental selective pressures that have contributed to modern human genetic and phenotypic variation (56, 100). Genomes evolve through processes including genetic selection and random drift that can alter the diet–disease relationship (66). Not all genes within the genome evolve at the same rate. Genes that are highly conserved among human populations and other species are those that typically encode proteins with essential functions that maintain life and are largely unaffected by the external environment. In contrast, rapidly evolving genes exhibit variation in DNA primary sequence across human populations that alter physiological function and contribute to human genetic and phenotypic variation. Such adaptive genes have historically permitted survival in specific environmental contexts. Hence, it is not surprising that genes involved in food, nutrition, and metabolism, as well as immune function, show some of the highest rates of gene evolution and hence genetic and phenotypic variation, as human populations that survived and expanded over time had to adapt to their somewhat unique local food and pathogenic environments (66). These adaptations enabled survival in a regional environmental context but can become maladapted and hence disease alleles when the environment changes, including changes in the food environment that resulted from the transition from hunter-gatherer to agrarian societies (21, 42).

Famine has been a common occurrence and selective pressure throughout human history, which has optimized biological function at the lowest dose of essential nutrients needed to maintain the species. The effect of this selective pressure is observed in humans and across other mammals through the interaction of cells with the nutrient environment, where binding affinities of essential nutrients for enzymes and transporters (Km, Kt) are highly similar among humans (and often among mammals) and conserved with minimal variation in requirements to maintain physiological function (97). Hence, the need for precision is more minimal when deriving Dietary Reference Intakes (DRIs) based on maintaining essential nutrient adequacy, because the need to establish population subgroups is limited to differences in physiological demands throughout the life cycle, as opposed to variation in physiology among healthy populations that are independent of life-cycle effects (e.g., genetics). There are a few exceptions, such as the impact of a common methylene-tetrahydrofolate reductase variant (*MTHFR* C677T) on cofactor binding leading to a higher folate requirement to maintain adequacy (96). However, our context has shifted from famine to an overabundance of foods, from addressing disease of nutrient deficiencies to addressing increasing rates of diet-related chronic diseases, all within the context of a food supply that is globalized in nature. As a result of our increased appreciation that there is meaningful heterogeneity in the diet–disease relationship, new approaches to establishing dietary recommendations become necessary, including new approaches to identifying and classifying subgroups (i.e., increasing precision).

**Dietary Reference Intakes (DRIs):**

a set of either cut points or ranges of nutrients and other food substances intake that are established by the Food and Nutrition Board at the National Academy of Medicine

## 3. HISTORY OF PRECISION IN NUTRIENT AND FOOD GUIDANCE

The prevention of nutrient deficiencies and subsequent deficiency-related disorders and the maintenance of physiological functions were the goals of the initial set of recommendations for intake of nutrients and other food substances (NOFS) (i.e., energy, fiber, and macro- and micronutrients) with specific reference ranges called the Recommended Dietary Allowances (RDAs) in the United States and the Recommended Nutrient Intakes (RNIs) in Canada. In the late 1990s, a harmonized framework for a set of recommendations that encompassed risk of both nutrient inadequacy and excess was put in place for both countries, known broadly as the DRIs (Table 2) (38). The DRIs are now established by a panel of scientific experts convened by the Food and Nutrition Board, part of the National Academies of Sciences, Engineering, and Medicine (NASEM), and represent recommendations for 22 population subgroups: two for infants; two for young children; six for boys and men; six for girls and nonpregnant, nonlactating women; three for pregnant women (depending on age group); and three for lactating women (also depending on age group). With this approach, population-based normative values are estimated on the basis of a distribution of requirements. However, it should be noted that the quantity and quality of the available scientific evidence vary from NOFS to NOFS, with evidence clearly lacking for some population subgroups (i.e., young children, pregnant women) and variability existing in the number of studies with sufficient sample sizes of high-quality design, as well as intermediary markers or adjudicated outcomes for risk of inadequacy and toxicity.

**Nutrients and other food substances (NOFS):**

a term employed by the DRI framework to indicate the difference between essential nutrients and nonessential, but relevant, food components, such as fiber

The DRI for energy (i.e., calories) intake is the Estimated Energy Requirement (EER) and represents estimation of the average caloric intake needed to maintain energy balance (in adults), as well as those for growth (in children) and for pregnant and lactating women, to support fetal growth and needs as well as those needed for the production of human milk, respectively (81). For macronutrients, specifically fat, carbohydrate, and protein intake, the DRI reference value is the Acceptable Macronutrient Distribution Range (AMDR), expressed as a range of recommended percent of energy intake that minimizes chronic disease risk while ensuring that the intake of essential nutrients avoids deficiency (39). For micronutrients (i.e., vitamins and minerals), the DRIs include an Estimated Average Requirement (EAR) to derive the population median requirement and an RDA that satisfies the needs for 98% of individuals in an apparently healthy population that are derived when scientific evidence is available to establish a clear end point for risk of inadequacy. Protein and carbohydrates also have an EAR and RDA in addition to the AMDR. When the scientific and experimental data are insufficient to estimate a direct relationship with an outcome, an Adequate Intake (AI) is set.An AI is generally derived on the basis of reported usual dietary intakes (from foods and beverages) from healthy populations, typically from national survey data. An AI is typically assumed to be higher than an RDA for most nutrients, but much less certainty around the AI exists. For many micronutrients, DRI reference values are based on risk of inadequacy or toxicity. The Tolerable Upper Intake Level (UL) is the highest level of a NOFS intake that is likely to pose no risk of adverse health effects to almost all individuals in the general population. The use of the DRIs in population-based food and nutrition policy and for individual planning purposes has been extensively reviewed elsewhere (8, 40). A further discussion of Chronic Disease Risk Reduction (CDRR) follows.

**Figure 1:**
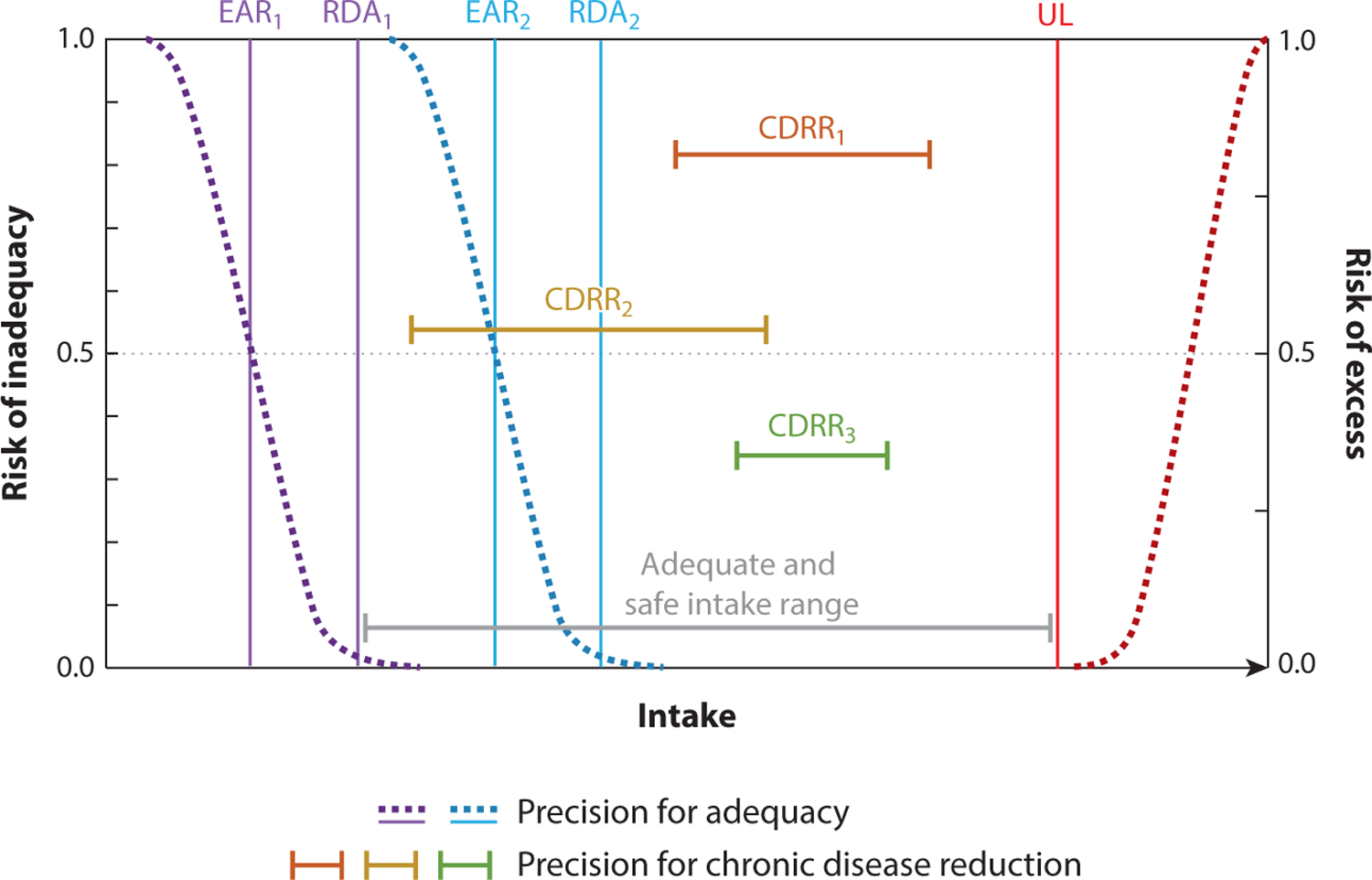
Dietary Reference Intakes (DRIs) taking population, precision, and personalized approaches. DRIs for nutrients and other food substances (NOFS) are established independently to maintain adequacy or risk of excess at the population and individual level [Estimated Average Requirement (EAR) and Recommended Daily Allowance (RDA)] and/or for Chronic Disease Risk Reduction (CDRR). Note that a CDRR can be lower than the RDA. The Adequate Intake (AI) does not have a consistent relationship like the EAR or the RDA and is therefore not presented in this figure; however, it is generally assumed that the AI value would be within the adequate and safe intake range (*gray horizontal bar*). The dashed curves indicate the level of risk (range between 0 and 1); dietary intakes that are within the range of the established RDA and the Tolerable Upper Intake Level (UL) are generally considered to be very low risk for most in a population group or at the individual level. Precision is introduced into the DRI process for essential nutrients when differences in requirements are identified currently within a subgroup on the basis of age, sex, or life stage (i.e., lactation and pregnancy), leading to separate recommendations for each subgroup group (RDA_1_, RDA_2_; EAR_1_, EAR_2_). DRIs set for chronic disease reduction for any given NOFS are expressed as ranges, unlike RDAs and EARs, which are discrete values. Precision is introduced into CDRRs (CDRR_1_, CDRR_2_, CDRR_3_) through identification of a population subgroup that is likely to respond similarity to a dietary exposure and by setting the end point for different or co-occurring disease states. Requirements that fall outside an adequate and safe intake level are very likely to require a more personalized approach than frameworks established to date. Some of this figure and caption language was adapted from Reference 38; copyright 2000 National Academy of Sciences.

Tailored guidance has always been a goal and outcome when establishing food- and nutrient-based guidance, but enhancing precision requires understanding, quantifying, and classifying variation in the needs in the population. The DRI framework first introduced the concept of precision guidance by acknowledging the existence of variation in the dose–response relationships for essential nutrients among a limited number of population subgroups (38). The need to consider only a limited number of population subgroups when establishing DRIs based on maintaining adequacy by specific subgroups is consistent with human natural history, given that less precision is required to prevent nutrient deficiency and toxicity states compared with chronic disease reduction (Figure 1). This is because most individuals within the healthy population tend to respond similarly to essential nutrient exposures and because nutrient exposures are the single root cause of deficiency diseases and manifest with specific symptoms across the population on a similar time course in healthy populations. For example, a diet deficient only in vitamin C will lead to early nonspecific symptoms of scurvy such as fatigue in approximately 4 weeks and to more specific, severe symptoms starting between 8 and 12 weeks including petechiae and corkscrew hairs. Similarly, toxicity responses to high doses often result in similar characteristics across the population; supraphysiological intake of zinc manifests in gastrointestinal symptoms and fatigue and may in turn initiate a copper deficiency (41).

While the DRI framework focuses specifically on NOFS of the diet, the Dietary Guidelines for Americans (DGA) have provided food- and beverage-based recommendations since 1980, with an early emphasis on specific food groups that has shifted more recently toward overall dietary patterns. The DGA represent a concerted effort on the part of the US federal government to provide a set of evidence-based dietary recommendations to “help promote health and prevent chronic disease” (105, p. 2; 106, p. 21). The food-based approaches, in particular dietary patterns research, have expanded the scope of the DGA and, in doing so, represent “quantities, proportions, variety or combination of different foods, drinks, and nutrients in diets, and the frequency with which they are habitually consumed” (108, p. 6).

**Dietary Guidelines for Americans (DGA):**

federal recommendations on food and beverage intakes issued every five years by the US Departments of Agriculture and Health and Human Services

Dietary patterns can be derived in various ways; all methods can be classified as independent or dependent on a particular health outcome (61).Outcome-dependent methods incorporate the outcome of interest or intermediate biomarkers into the models used to derive patterns; examples include reduced rank regression and classification and regression tree analysis. Although these methods are useful for examining the relationship between diet and a particular outcome, most nutrition researchers utilize methods agnostic to the outcome of interest to describe general diet quality. There are two broad classes of methods for developing outcome-independent dietary patterns: data-driven techniques such as factor analysis or cluster analysis, which emphasize data reduction techniques or clustering individuals based on their reported dietary intakes, and indexed-based methods, which are based on a priori patterns according to dietary guidelines or recommendations. Classifying dietary patterns through data-driven approaches can lead to multiple subjective decisions on the part of the researcher to derive them, complicating comparisons of patterns in different cohorts or population groups and reducing their utility in research for defining food-based patterns. However, both factor and cluster analysis are useful data-reduction techniques to determine the underlying structure within a complicated data set, as is the case with dietary exposures. The use of indexes and scores essentially creates a report card for how well a diet conforms to a predefined rubric. While subjectivity exists in how the rubric is developed, this method provides a standardized framework to compare across studies. For this reason, the 2015 Dietary Guidelines for Americans Advisory Committee (DGAC), building on systematic reviews (108), concluded that indexes and scores are a preferred method to capture dietary patterns and the complexity of the entire diet. Various indexes and scores exist (75, 87) such as the Healthy Eating Index (52, 53, 60, 88), the Mediterranean diet score (86, 103), and the Dietary Approaches to Stop Hypertension (DASH) score (71). Using this framework, the 2020 DGAC concluded that strong evidence exists for higher-quality dietary patterns that are associated with lower risk of all-cause mortality (33) and cardiovascular disease (28) and that moderate evidence exists for dietary patterns that are associated with type 2 diabetes (12), bone health, overweight and obesity (11), and colorectal and breast cancer in adults (13).

The most recent 2020–2025 DGA took a life-stage approach to evaluating the available scientific evidence including all life stages, with an additional emphasis on focusing on the unique nutritional needs during pregnancy and lactation and for infants and toddlers, birth to 24 months, in addition to recommendations for all Americans aged 2 years and older. Much less scientific evidence was available to evaluate dietary patterns and various health outcomes during pregnancy and lactation and among young children.

In addition to recommended food patterns to follow, the DGA Scientific Advisory Committee (29) and the DGA (107) also identified life-stage-specific NOFS of public health relevance and public health concern across all life stages for the first time, guided by a proposed framework including dietary exposures, biological end points, or prevalence of disease or validated surrogate markers of disease (4).Thus, while some dietary guidelines are universal, a need was recognized to expand specific recommendations to the population on the basis of life stage—this represents the first public health nutrition approach targeting specific groups on the basis of life stage beyond that of the DRIs. The DGA also recognize that there are multiple potential dietary patterns that exist for similar health outcomes, such as the Mediterranean diet or vegetarian patterns for the prevention of cardiovascular disease. The dietary patterns reviewed by and included in the DGA do not represent the use of dietary supplements, substantially underestimating nutrient exposures for the half of adults and one-third of children that use dietary supplements (5–7).For this reason, a Total Nutrient Index, inclusive of nutrient exposures from supplements, in addition to those from foods and beverages, has been developed to be used in conjunction with food- and beverage-based indexes to improve the comprehensiveness of exposure classification (23, 24).

## 4. CHRONIC DISEASE IN NUTRIENT AND FOOD GUIDANCE

Today, most Americans have one or more chronic diseases and related factors such as medication use (98) that can alter nutrient requirements leading to deficiency and secondary comorbid diseases. At present, six in ten Americans have one chronic condition and four in ten Americans have two or more chronic conditions (29). More than 70% of Americans have overweight or obesity, and the prevalence of severe obesity has increased over the past two decades (29). The high rates of overweight and obesity are an important public health problem in and of themselves but also increase the risk for cardiometabolic disorders and some types of cancer.

The disease process is known to influence nutrient absorption, catabolism, and nutrient partitioning among tissues, likely leading to differences in requirements to maintain adequacy for some key nutrients (99).The term special nutrient requirements refers to nutrient requirements needed to maintain adequacy in disease states but has not been well developed beyond clinical case studies (99). Chronic diseases, genetic diseases including inborn errors of metabolism, inflammation, dietary intolerances, medications, allergies, trauma, and infection, among other pathologic states, can alter essential nutrient requirements, in terms of both deficiency and toxicity, but disease, its etiology, and comorbidities are not considered in the current DRI framework as it pertains to the apparently healthy population as opposed to clinical populations (99). These differential requirements for NOFS are currently considered under medical nutrition therapy and represent a more personalized guidance that currently is beyond the scope of the DRIs.

The biological underpinnings for developing DRIs aimed at chronic disease reduction do not support restricting nutrient- and food-based recommendations to apparently healthy individuals, because there are no firm diagnostic criteria for when a disease begins. Chronic diseases initiate and manifest throughout the entire life span and are tightly linked to aging as well as to numerous static and dynamic factors and environmental exposures, including food. The gradual decay of biological systems is a hallmark of both aging and chronic disease progression that start at the earliest stages of life (64). Biological network and system decay lead to erosion of function and/or increases in stochastic behavior leading to increased variability/stability in network outputs and system behavior that becomes incompatible with health (65). For example, at the molecular level, this can be quantified by the erosion of epigenetic landscapes across the human genome leading to alterations in gene expression patterns and network function (65) and age-related changes in plasma metabolites (78), some of which are biomarkers of nutritional status, and changes in redox potential (94).

Numerous lifestyle, environmental, and intrinsic physiological risk factors all contribute and interact to affect rates of biological aging and the initiation and progression of chronic diseases, including certain cancers, type 2 diabetes mellitus, and cardiometabolic and neurodegenerative diseases, among others (10, 37, 76, 90). Family history (i.e., genetics) is the dominant nonmodifiable predictor of life span (47). Chronic disease initiates during the earliest stages of human development through mechanisms that include genome mutations and epigenetic programming of stem cells (83). In this regard, nutrition requirements can be seen as a dynamic countermeasure to slow and/or maintain age-related declines in the functional capacity of biological systems and networks needed to promote health, and this is where precision nutrition efforts could be most beneficial. Population-based approaches to decrease biological aging will require better linkage of biomarkers of nutrient exposure, status, and function to biomarkers of disease and aging.

## 5. CHRONIC DISEASE REDUCTION CONSIDERATIONS

**Figure 2:**
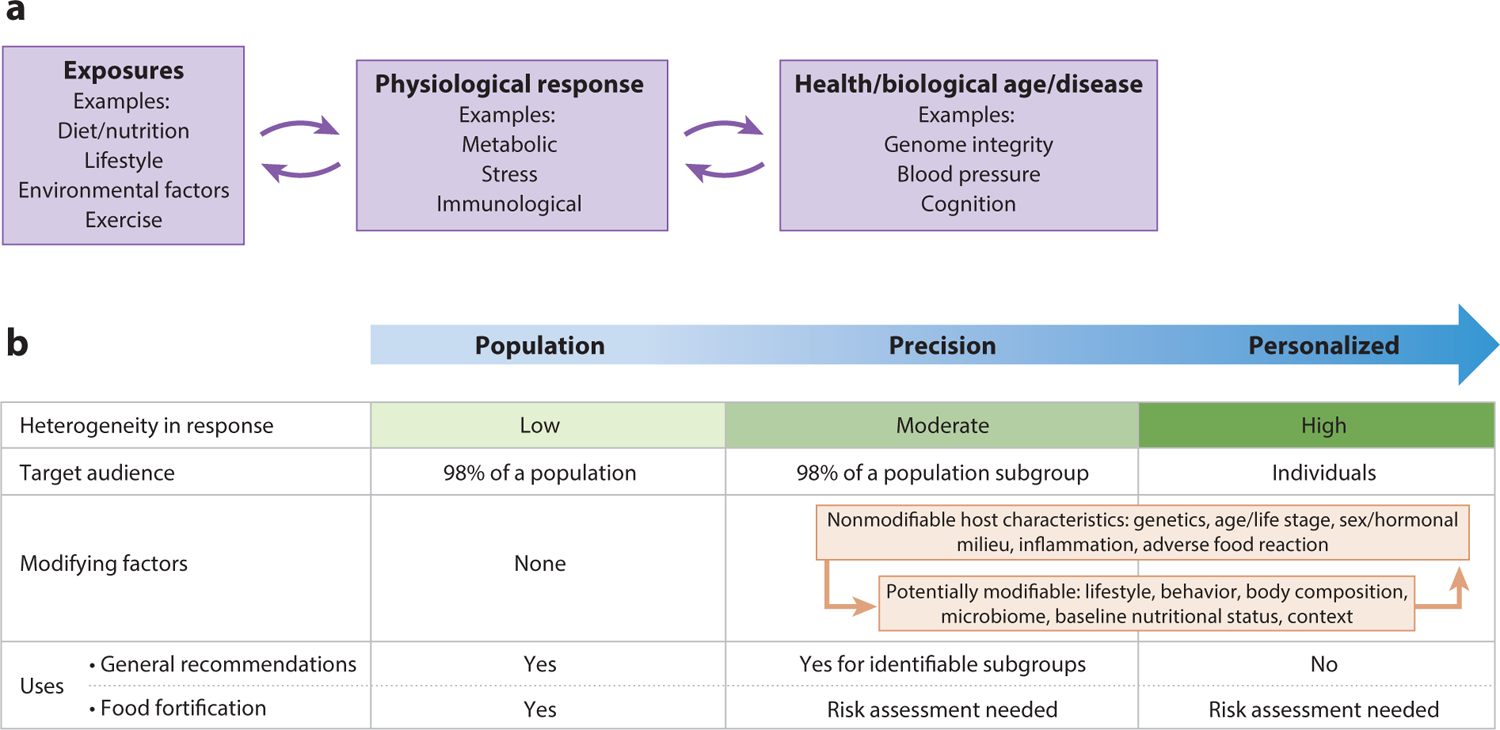
(*a*) Biomarkers linking exposures to disease. Implementing precision nutrition requires knowledge, tools, and measures (e.g., biomarkers) that quantify and connect exposures to physiological responses that influence health, disease, or validated surrogate markers. It is important to note that the connection between exposures and physiological response is reciprocal through feedback loops (e.g., diet can affect inflammation, which in turn affects dietary needs), and that physiological responses are reciprocal with health/disease/aging. (*b*) The relationships among and use of population, precision, and personalized nutrition approaches. Modifying factors that drive precision include those factors that are intrinsic to the host (e.g., genetics/ancestral history, age) and modifiable and dynamic factors that may change across the life course (e.g., physical activity, sleep, stress, microbiome). Precision nutrition is the classification of modifiable factors that alone or in combination with fixed factors may lead to a differential metabolic response to dietary exposures. Additionally, there are interactions between and among these factors, which add complexity to the precision nutrition lens and are much less predictable and quantifiable than each factor in isolation.

In 2017, the NASEM developed a framework to formally incorporate CDRR values into the DRIs. That change emphasizes a shift in dietary guidance toward health promotion and reduction of chronic disease risk in addition to avoiding inadequacy (117). This paradigm shift to reducing chronic disease risk through diet and its rationale are published elsewhere (116, 117). Unlike diseases of deficiency, most chronic diseases manifest over time and result from cumulative effects of the aging process and behavioral and lifestyle factors, as described above (50). Establishing food and nutrient intake recommendations for chronic disease reduction requires consideration of multiple factors that independently and interactively contribute. These additional biological factors increase population heterogeneity in the diet–disease relationship, further driving the need for greater precision in dietary recommendations. Implementing precision nutrition requires knowledge and tools (e.g., biomarkers) that quantify and connect exposures (e.g., diet/nutrition, lifestyle, environmental factors, exercise) to physiological responses (i.e., metabolic, stress, immunological) to health and disease (e.g., genome integrity, blood pressure, cognition). It is important to note that the connection between exposures and physiological response is reciprocal through feedback loops (e.g., diet can affect inflammation, which in turn affects dietary needs), and that physiological responses are reciprocal with health, disease, and aging (Figure 2a). The causes and modifiers of the diet–chronic disease relationship include but go far beyond the role played by essential nutrients in maintaining metabolic and other functions. NOFS can influence chronic disease onset and progression through (*a*) secondary pathogenic effects of essential nutrient deficiencies and excesses; (*b*) pathogenic effects of imbalances among essential nutrient intakes; (*c*) pathogenic effects of oxidative stress, immunological responses, and other responses to exposure to particular food components; (*d*) intake of nonessential bioactive food components that influence chronic disease in the absence of essentiality or toxicity; and (*e*) eating behaviors including temporal eating patterns, otherwise referred to as chrono-nutrition (36).

### 5.1. Essential Nutrient Deficiencies and Excesses

Diseases resulting from essential nutrient deficiencies and toxicities have been clinically recognized and well characterized (15) and have historically been considered in the process for establishing DRIs focused on maintaining nutritional adequacy and physiological function (112). When DRIs are extended to include chronic disease risk reduction, other physiological responses to nutrients outside their known functional roles must be considered (Figure 2b). The diet–chronic disease relationships transcend physiological function and extend to immunological and stress responses to dietary components, and these responses can either reduce or increase disease risk. It is increasingly recognized that common asymptotic, subclinical inadequacy of certain essential nutrients can be associated with markers of increased chronic disease risk that often cannot be attributed directly to their known physiological function. Both essential nutrient deficiencies and excesses can cause inflammation, and elevated status of certain nutrients can alter physiological processes that can increase or decrease risk for a chronic disease without causing toxicity. For example, in animal models, marginal magnesium deficiency stimulates oxidative stress and secretion of proinflammatory mediators from phagocytic cells, resulting in chronic inflammation. In human populations, dietary magnesium intake is inversely associated with cardiometabolic disease, metabolic syndrome, and colorectal cancer, as well as serum or plasma C-reactive protein (CRP). CRP is a biomarker of inflammation and is a risk factor for many chronic diseases (84, 85). Inflammatory mediators, including chemokines and CRP, serve as biomarkers that report on aging, exercise, nutrition, and chronic diseases including atherosclerosis, diabetes, obesity, sarcopenia, and Alzheimer’s disease (19). Nuclear factor kappa B (NF-κB) serves as a central mediator that connects inflammation to nutrition and aging by regulating proinflammatory mediators including CRP, tumor necrosis factors α and β, interleukins (IL-1β, IL-2, and IL-6), and chemokines (IL-8) (20). Similarly, subclinical vitamin C inadequacy has been associated with inflammation, elevated CRP levels, and depressed immune function (27). On the other hand, high intake of essential nutrients in the absence of nutritional deficiency may lower inflammation in those with existing chronic disease. Vitamin D supplementation may decrease blood CRP levels in children with overweight and obesity (51) and improve inflammatory markers in pediatric intestinal bowel syndrome (95). Both folate and vitamin B_12_ deficiency have been shown to exacerbate inflammation associated with disease and infection (58, 109), whereas folic acid supplements have been shown to lower CRP blood levels (2). Overall, our understanding of the role of essential nutrient deficiencies and excesses in oxidative stress and inflammation is limited. However, these illustrative examples demonstrate the need to further develop and extend the fundamental knowledge and clinical evidence base in the nutrient–chronic disease relationship that transcends metabolic, signaling, and other essential functions, which are the foundation of current DRIs developed to maintain adequacy.

### 5.2 Imbalances Among Essential Nutrient Intakes

The multifactorial etiology of chronic disease initiation and progression is characterized by interactions among intrinsic biological systems and extrinsic environmental factors, including essential nutrients, that influence the function of physiological systems that are critical to maintain health. Virtually all metabolic, signaling, and other physiological networks involve interactions among multiple essential nutrients, and imbalances in nutritional status among nutrients in the same system have been linked to accelerating and/or exacerbating chronic disease. Sodium, potassium, and chloride play essential roles as electrolytes that regulate fluid balance in cells and play a key role in maintaining blood pressure. Imbalances in the potassium/sodium ratio in urine reflect dietary exposures and are associated with an increased risk for hypertension (113) and cardiovascular disease (59) in adults, as well as morbidity in preterm infants (44, 92). Although population guidance suggests sodium intakes should be within the recommended intake range (on the basis of age and sex), many populations are not impacted by the effects of sodium and blood pressure. While some individuals display no association between the amount of dietary sodium consumed and blood pressure, some population subgroups are considered to be salt sensitive and are more likely to respond negatively to higher sodium in terms of blood pressure on the basis of age, sex, and ancestry, as well as those with compromised kidney function, obesity, and existing hypertension (9, 32, 55, 82). However, looking at the role of sodium in isolation can be misleading; the dietary ratio of sodium to potassium is critical to the impact it has on hypertension. Other minerals as well as interactions with dietary patterns are also related, making precision guidance quite challenging. For example, the DASH-Sodium randomized clinical trial looked at a healthy dietary pattern intervention compared with a typical American diet with three different levels of dietary sodium: low, intermediate, and high (101). On the control diet, the differences among the three sodium arms were consistently dose dependent and consistent across those subgroups known to be at higher risk; however, the magnitude and significance of the sodium effect were attenuated when the DASH diet, compared with the American diet, was being followed, suggesting that a healthy dietary pattern can offset the negative effects of sodium on blood pressure in high-risk population subgroups. As a second example, there are concerns in the literature that imbalances in the status of the B vitamins folate and vitamin B_12_ and their interactions can be pathogenic. Folate-mediated one-carbon metabolism is a metabolic network necessary for synthesizing nucleotide precursors and remethylating homocysteine to methionine, which supports more than 100 cellular methylation reactions (34). The network requires many essential micronutrients including vitamin B_12_, vitamin B_6_, folate, niacin, and riboflavin. Elevated folate status in the context of vitamin B_12_ deficiency has been associated with exacerbating the neurological, metabolic, and clinical manifestations of vitamin B_12_ deficiency alone (73), although no mechanisms indicating a causal relationship have been identified (26, 68). Nonetheless, these potential deleterious interactions have raised concerns regarding excess intake of folic acid (35).These examples emphasize the need to consider and recommend nutrient status ranges (Figure 1) in the population that promote health, by optimizing nutrient–nutrient interactions within a given biological network, leading to chronic disease prevention, adding yet another layer of complexity in our ability to make more precision guidance.

### 5.3 Stress and Immunological Responses to Food Components

Food intolerances and food allergies are, respectively, nonimmune and immune adverse reactions to food (45). They are common inflammatory chronic diseases, their prevalence may be increasing, they impact quality of life, and they are associated with higher risk for other chronic diseases (3). It is estimated that up to 20% of individuals exhibit gastrointestinal food intolerances (45, 63). There are many causes of food intolerances including: (*a*) pharmacological effects of dietary components such as short-chain fermentable carbohydrates, otherwise known as fermentable oligo-, di-, monosaccharides and polyols (FODMAPs); (*b*) nonimmunologic gluten sensitivity; and (*c*) enzyme and transport defects (63). They are generally managed through exclusion diets. The most common clinical presentation of adverse food reactions is irritable bowel syndrome (IBS), which increases risk for gastrointestinal cancers, but adverse food reactions can also negatively affect the cutaneous, respiratory, neurological, and cardiovascular systems (45) and increase risk of breast cancer (16).

Food allergies are distinct from other forms of food intolerances, and sometimes the same dietary component can trigger multiple mechanisms of intolerance (110). Food allergies occur when there is an immunoglobulin E (IgE)-mediated immunological response to antigenic epitopes present within specific food components (45).Other food antigens can mediate immune and inflammatory responses. Data science technologies have revealed increased IgG antibody reactivity to epitopes present in foods in patient populations, with the most common reactive foods being casein, cow’s milk, wheat, gliadin, egg whites, and rice; less common is reactivity to nuts, vegetables, fish, seafood, and meat products (22). For example, celiac disease is a genetically linked autoimmune enteropathy that sensitizes individuals to the gliadin and glutenin proteins in gluten, which is present in certain grains, resulting in an inflammatory response. It manifests in approximately 1% of the population (69). Gluten intolerance, on the other hand, is more common, affecting up to 6% of the population, and, overall, nonceliac wheat sensitivity may affect 10% of individuals (48). Gluten intolerance is not genetically linked, nor does it trigger an allergic response, but it can present with similar symptoms as celiac disease, due to activation of both the innate immune system and multiple inflammatory pathways by a component of gluten (18). Other wheat proteins known as amylase trypsin inhibitors (ATIs) (48) also activate the innate immune system and contribute to overall wheat sensitivity (18).

There are multiple other adverse reactions to food and food components that are independent of immune involvement and manifest through many distinct known and unknown mechanisms. They are classified as either host dependent or host independent (45). The most common clinical manifestations include urticaria or angioedema but also include asthma, gastrointestinal (GI) symptoms, hypotension, headache, and eczema. Nonimmune, host-independent food intolerances involve chemicals with pharmacological activity in food that affect sensitized individuals and include salicylates, vasoactive amines (e.g., histamine), glutamates (e.g., monosodium glutamate), and caffeine but their etiology and management remain elusive (63).Nonimmune, host-dependent food intolerances generally involve a lack of host metabolic capacity as seen in lactose and fructose intolerance as well as nonspecific reactions to certain foods including FODMAPs. These compounds elicit osmotic effects in the GI tract, encourage undesirable fermentation by colonic bacteria, and can serve as prebiotics that can negatively alter the composition of the microbiota, trigger inflammation, and induce IBS symptoms (45).

### 5.4 Intake of Nonessential Bioactive Food Components That Influence Chronic Disease in the Absence of Essentiality or Toxicity

The NASEM framework for developing DRIs based on chronic disease risk reduction (79) recognizes that intake of nonessential bioactive dietary components, otherwise known as xenobiotics, has the potential to reduce chronic disease risk, and therefore can be evaluated in the process for establishing DRIs (114). This include bioactives such as the nonprovitamin A carotenoids lutein and zeaxanthin, which have been associated with eye health and eye development (49), flavonoids and other polyphenols that have antioxidant and transcriptional activation activity and have been associated with protection of several chronic conditions (77), and potentially semiessential nutrients such as omega-3 fatty acids. For example, omega-3 fatty acid intake has been associated with cardiovascular disease risk factors (54, 91, 93, 120), cognitive function (74, 111), risk of depression (1), and preterm birth (72), among other outcomes (67, 119). However, data supporting these associations are not consistent in the literature, which may speak to potential pockets of the population that may have improved health outcomes when differential intake ranges are consumed. Because these compounds can support health but are not technically essential for life, greater population heterogeneity in their biological and health effects compared with essential nutrients is expected due to variability in their historic regional abundance and different selective pressures that were likely operative. Furthermore, cellular concentration of xenobiotics, as well as many synthetic pharmaceutical agents, is regulated by their catabolism; these substrates are degraded or bioactivated by cytochrome P450s, which tend to exhibit wide variation in substrate specificity and catalytic activity within and among human populations, leading to heterogeneity in functional responses to various substrates (70).More information concerning the role of bioactive food component intake in chronic disease reduction, and population variability in the health effects of bioactives, will require building the evidence base for establishing recommended intake ranges that are likely to vary on the basis of the end point under consideration, as depicted in Figure 1.

### 5.5. Eating Behaviors Including Temporal Eating Patterns, Otherwise Referred to as Chrono-Nutrition

**Figure 3:**
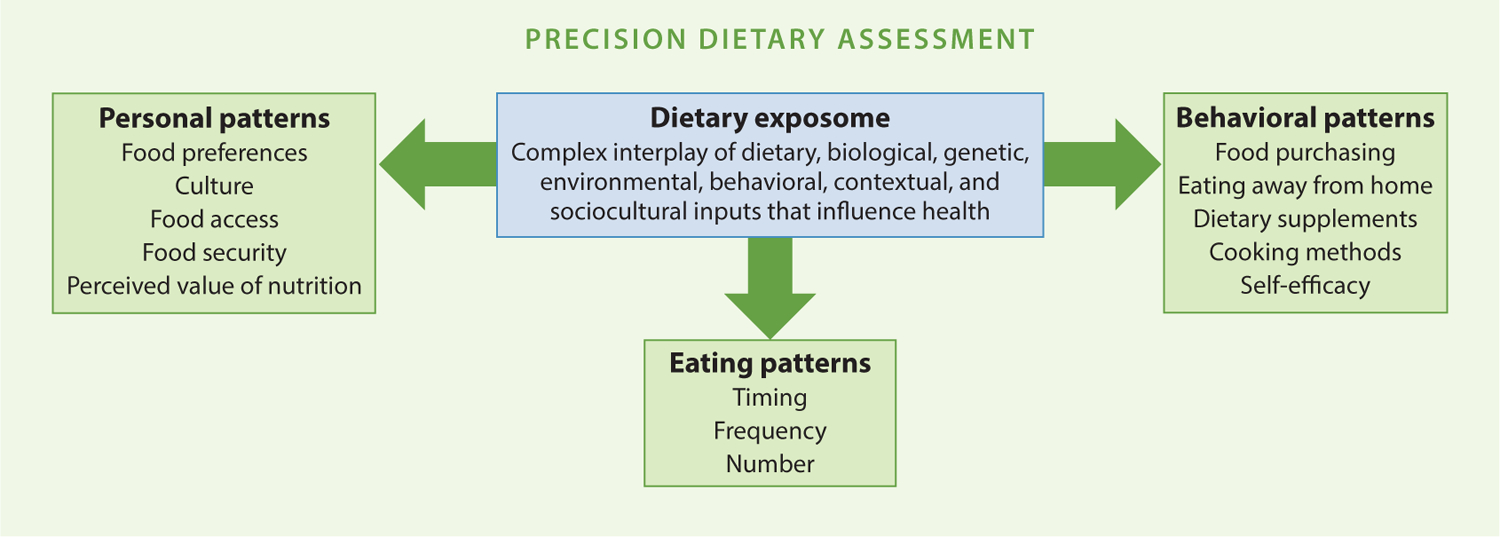
Defining the dietary exposome and additional factors that will need to be captured to improve precision nutrition guidance. Some of the many example factors (i.e., components of personal, eating, and behavioral patterns) of interest that are critical to capture or consider as part of improved dietary assessment methods are shown; please note that this example is not exhaustive of the multitude of factors that may be relevant for research and monitoring purposes.

Food- and nutrient-based recommendations focus on what and how much of a particular food or nutrient should be consumed, but not when they should be consumed (17). There is increased recognition that the timing and frequency of eating within a day can affect health outcomes (36, 102). It is also recognized that both time of eating and direct biological effects of certain NOFS, including caffeine and polyphenols, can modify circadian clocks. The interaction of NOFS with physiological circadian rhythms, fasting (including frequency of daily eating), and other eating behaviors all impact metabolic process and contribute to the diet–disease relationship. Furthermore, they show interindividual variability in physiological responses and are currently not considered within the scope of the DRI or DGA processes but are an important dimension in the dietary exposome depicted in Figure 3. Capturing not only what people eat but also these contextual factors of food behaviors will be critical to understanding how to tailor precision nutrition recommendations. Research into precision dietary assessment to capture these and other contextual factors is needed to advance our ability to make more precise dietary advice.

## 6. EVIDENCE NEEDS AND DATA GAPS TO BRING A PRECISION NUTRITION LENS TO NUTRIENT AND FOOD GUIDANCE FOR CHRONIC DISEASE REDUCTION

Unlike nutrient- and food-based recommendations aimed at preventing nutrient deficiencies, those aimed at reducing chronic disease must consider not only the causal relationship(s) between diet and disease but also the contribution of diet to overall chronic disease risk, relative to other lifestyle interventions. Paramount is the need for common evidentiary standards across all individual lifestyle risk factors to inform the most effective behavioral modifications at the population and individual levels to achieve chronic disease reduction. Thus, to reduce risk of disease and/or to optimize human health, our dietary recommendations should be more responsive to the unique nutritional needs of various population subgroups with known differences in responses or with similar risk levels or behavioral patterns and their relative impact compared with other lifestyle modifications.

Establishing DRIs for chronic disease risk reduction requires strong diet–disease links with adjudicated end points or validated surrogate biomarkers of disease risk that can be tied back to lifestyle modifications, including diet (Figure 2a) (116). As such, for the first time, a unique feature of the DRI for sodium is a shift from prevention of potential deficiency and excess to identifying the optimal dietary intakes for reduced risk of chronic disease with the creation of the CDRR. The CDRR can be set when there is, at a minimum, a moderate degree of causal evidence between a dietary intake level and a health outcome. Currently, the CDRR is established only for sodium, on the basis of a moderate strength of evidence for cardiovascular disease and hypertension, integrated with strong evidence for sodium and blood pressure regulation (80).

## 7. ADDRESSING THE DATA GAP WHILE MAINTAINING PUBLIC TRUST

In many respects, deriving DRIs for most nutrients is aspirational with today’s current knowledge and approaches, but there is still a need for working toward increased accuracy and precision where possible and supported by evidence. A lack of clinically meaningful end points or validated surrogate biomarkers for most chronic diseases and studies establishing their causal relationships with nutritional exposures creates uncertainty and prevents major progress. For almost all NOFS with an existing DRI, there exists a dearth of data from the kind of high-quality research studies needed to best optimize the diverse needs of our population. Furthermore, while the approach of an isolated assessment of one nutrient and one end point enabled the development of DRIs for maintaining adequacy, it is unlikely to advance our understanding of the complexity of dietary intakes with respect to chronic disease reduction. NOFS have antagonistic and synergistic interactions with other NOFS that must be accounted for in examining their bioavailability and function in humans. To date, this complexity has not been adequately measured or accounted for in research studies, though multi-omics approaches are advancing our understanding of NOFS interactions. The prediction and understanding of differential responses to nutritional exposures culminate in risk disparities for diet-related chronic disease based on population subgroup characteristics such as sex, age, genetics/epigenetics, disease, and environment and behavioral factors, to name just a few.

The urgency to address the increasing rates of chronic disease through developing food and nutrition guidance for the public must be balanced by clearly and transparently communicating the strength of the scientific evidence supporting food and nutrient recommendations for chronic disease reduction to garner public trust and uptake of the recommendations. At present, most Americans do not follow the DGA (104). Major changes in recommendations over time have eroded public trust, and public confidence in nutrition research and population-based dietary recommendations needs considerable strengthening (43, 46). Public acceptance of food and nutrition guidance is essential for the aspirational goal of achieving public health through precision nutrition.

## 8. SUMMARY: PRECISION NUTRITION AND PUBLIC HEALTH RECOMMENDATIONS, FOOD POLICY, AND DIETARY PATTERNS

The concept of precision nutrition is not new to nutrition guidance, though the terminology is novel (89). Yet at the same time, despite all our scientific advances and efforts to improve health through nutrition, our nations face an unprecedented burden of diet-related chronic disease, increased prevalence of overweight and obesity starting in young children, and declines in life expectancy that are not evenly distributed among racial-ethnic groups (29, 31). While largely accepted scientifically, targeted dietary guidance toward primary prevention of chronic disease is recognized as more beneficial than treatment and management of chronic disease. However, the concept of precision and personalized nutrition today is still aspirational due to evidence gaps and public acceptance of population-based guidance in general. How to actualize more targeted guidance has been difficult, if not impossible, to date.

Decades of research have demonstrated that there are population subgroups with differential responses to nutrients or diets, to habitual diet, or to dietary interventions with respect to physiological effects and health outcomes (i.e., so-called responders and nonresponders) (89). Nevertheless, with few exceptions, public health nutrition guidance for chronic disease reduction previously focused on a one-size-fits-all type of approach, informed by systematic reviews of the available literature based on the premise that the majority of the population responds similarly; that premise has been successful for some end points of relevance (e.g., deficiency), but we have largely, as a society, moved beyond deficiency disorders. We have yet to actualize the potential of tailoring precision recommendations for diets and consumption of NOFS at the population level focused on chronic disease reduction. The omics-based approaches have affirmed inherent individual differences in our genetics, metabolism, and response to environmental and lifestyle changes and have extended our fundamental knowledge of biology, but the costs of translating these data for classifying responders and nonresponders with respect to diet and nutrition for public health improvement are prohibitive and not practical at this time.

The use of public health nutrition recommendations extends well beyond developing guidance to promote health; recommendations also inform food fortification programs, both mandatory and voluntary. Implementing precision nutrition into food and nutrition policy represents an even greater challenge in improving the alignment across food, diets, and health. Most fortification programs are intended for broad-based coverage for all of the population and informed by population surveillance data of nutritional deficiency status (e.g., fortification of salt with iodine; vitamin D added to dairy products) or replacements of nutrients lost in the processing of foods. However, fortification of grains with folic acid for the prevention of neural tube defects (NTDs) reflects the first population nutrition coverage approach intended to achieve a health outcome for a narrow target population: reproductive aged women who are at risk for having a child with an NTD and who need additional folic acid to mitigate this risk (25). This amounts to targeting approximately 2,500 births per year. Folic acid fortification policies are grounded by convincing data from randomized, controlled trials that folic acid could prevent the occurrence and recurrence of NTD-affected pregnancies. At the time of its implementation, there was no clear understanding that additional folic acid would benefit most of the nontarget population, other than reducing the risk of low serum and red blood cell folate. Folic acid fortification is a hybrid of population and precision nutrition decision-making at the policy level and was achieved only because there was no evidence of harm with respect to the fortification level to the general population in addressing the nutrition needs of a small subset of women. Because the benefit of folic acid fortification was realized by a small percentage of the population, there was substantial pushback based on hypothetical but not evidence-based concerns of potential harm to other subgroups in the population (35, 68). Nevertheless, the program has been one of the most successful public health interventions in reducing health risks for the target population, with no actionable evidence of adverse effects documented for nontarget populations. Continued monitoring of both beneficial and adverse effects of folic acid fortification remains essential (30,118).This example clearly illustrates the tension that exists when population-based approaches designed to reduce the risk of a diet/nutrient-related pathology are implemented despite not everyone in the population being at risk or able to respond to the intervention. The aspiration to achieve precision will be possible only when women at risk for having an affected child can be identified and classified with confidence and there is timely and effective implementation of the intervention.

Herein we reviewed the complex and multifactorial biological underpinnings of optimal nutrition from a theoretical perspective. We recognize the efforts of the DRIs and DGA to garner the most apt and current dietary recommendations to reduce risk of chronic disease. Given the high proportion of the population that experiences chronic disease and its associated physical, mental, and financial sequalae, a need exists to make more specific and actionable recommendations to population subgroups, and improve the food supply, to promote health and reduce the risk of diet-related chronic diseases. The experience with folic acid fortification illustrates that this goal is achievable.

In many respects, deriving such authoritative recommendations is arguably aspirational with today’s current knowledge and approaches. Data gaps and a lack of clinically meaningful endpoints or validated surrogate biomarkers for most chronic diseases with complex etiologies and their relationships with nutritional exposures prevent major progress. Furthermore, while the approach of an isolated assessment of one nutrient and one end point enabled the development of DRIs for maintaining adequacy, it is unlikely to advance our understanding of the complexity of dietary intakes with respect to chronic disease reduction as system approaches are needed. Moreover, given the long latency of most chronic diseases (or their validated surrogate bioindicators), the research on and the association between chronic disease and diet tend to be observational in nature, a weaker form of evidence that makes it difficult to establish cause and effect (i.e., exposure and outcome) relationships. Historically, scientific evidence on relationships between nutrient and other food component or dietary pattern exposures and chronic disease risk has been included in the scientific review process for existing guidance; the data were difficult, if not impossible, to meaningfully use because of the large variations in susceptibility to risk. But it is the very nature of these variations in response that led to the field of precision nutrition. Thus, to reduce risk of chronic disease or to optimize health, dietary recommendations should be even more responsive to the unique nutritional needs of various population subgroups with known differences in responses, whether those are based on similar risk levels, clusters of traits, outcomes of importance, and/or behavioral patterns that remain to be seen. As a scientific community, how do we meaningfully actualize the concept of precision nutrition with urgency without overpromising the public with recommendations based on weak evidence that do not stand the test of time?

Precision nutrition guidance focuses on the space between the person and the population. A population subgroup may be defined by a wide array of factors such as sex, gender, age, responders/nonresponders, shared ancestry, disease, clustered metabolic traits, and so forth. This requires a literature base to support such recommendations, and to date no authoritative bodies have made precision nutrition recommendations for chronic disease reduction, other than those based on age and life stage as previously mentioned, although the enthusiasm for the concept is shared by the US federal government in terms of the funding for the US National Institutes of Health (NIH) All of Us study and other US Department of Agriculture funding targeted toward more focused dietary guidance. Without thoughtful planning and guidance, how can these financial outputs be matched with scientific inputs to address the number of barriers that need to be resolved to evolve nutrition science in meaningful ways to promote health?

First, we wildly lack the needed types of data to inform precision nutrition efforts, a lack that in some cases is driven by structural barriers. Existing funding mechanisms are fueled by 5-year competitive cycles. Chronic diseases do not occur on this federally mandated timeline; rather, longitudinal studies, with multidisciplinary teams, are needed to resolve the cumulative complexity of diet with outcomes. The diet–chronic disease relationship can also transcend time and physiological function and extend to immunological and stress responses, among a multitude of others, to dietary components, and these responses can either reduce or increase disease risk. The diet–chronic disease relationship is modified with interactions among diet and most other lifestyle behaviors. Because the antecedents of chronic disease and its progression are so multifactorial, considerable heterogeneity in response to different dietary patterns and nutrients among individuals and population subgroups exists, coupled with the complex interplay of foods and nutrients in the diet. Next, there is rarely one outcome/health condition of interest/concern; how do we truly decide how to provide guidance to reduce risk of cardiovascular disease when many other metabolic and diet-related diseases are likely to co-occur? This forces, and not unnecessarily, our research communities to focus on end points, which are often clustered, but we are trained to think in silos and not in the multidimensionality of reality. Second, while we do indeed have some longitudinal data, nutrition researchers are woefully untrained to capitalize on the advances in machine learning and artificial intelligence that have been so powerful in other fields, such as environmental health, and that could capitalize on the existing data that are relevant to help inform precision nutrition efforts. Moreover, we are faced with ever-evolving advances in factors that may mediate the relationship between exposure and outcome such as the microbiome. The microbiome, and its importance, remains elusive, limiting its current usefulness when developing recommendations. Given that the host microbiome can be easily manipulated within days with diet and within hours with antibiotics, have we put too much reliance on its role in how nutrition modulates the host response?

Now is the time to pause—now is the time to collect the requisite data—now is the time to admit that we know next to nothing about how to implement precision nutrition. We need investment in better methods to assess exposure and then to assess how that exposure manifests in disease. Currently, we have weak methods for exposure assessment, and as a scientific community we suspend disbelief on the black box between exposure and outcome. The first step in precision nutrition is precision dietary assessment (Figure 3): We must know much more than what factors need to be captured; we must also understand the context of food behaviors if our recommendations are to be actionable and meaningful. Quantifying not only what we eat but also the context, timing, food preferences, cultural practices, agency, access, and so forth all must be implicit in moving the needle on food behavior changes. Finally, real-time monitoring, data science, and integrated efforts across disciplines will be the only reasonable solution.

## Figures and Tables

**Table 1:** Proposed key terms to describe levels of nutrition guidance^a^

Term	Target	Definition	Optimized	Examples
Personalized	Individuals	Specific dietary recommendations provided on the person level, usually based on intrinsic host biology including genetic predisposition, microbiome, and immunological response, among others	When genetic or other unique biological or physiological factors cause a differential need for consumption or avoidance of nutrients or food substances or foods/beverages based on host response	Lactose intolerance; celiac disease; phenylketonuria; food allergies and intolerance; responders and nonresponders
Precision	Population subgroups	Specific tailored dietary recommendations that exist on a distribution of risk profiles or on the bases of growth, development, or life stage	Risk profiles that predispose to end points that can be ameliorated or managed by diet among identifiable subgroups	Sex, age, and life-stage groups; those at risk for type 2 diabetes
Public health	Entire population	Broad-based guidance to most in the population	When most people react in similar and predictable ways and low risk of harm exists	Fortification programs to prevent deficiency disorders; strong scientific agreement

a These definitions put forth are not the first to try to describe the nuances of these terms, and our proposed list builds off the original work of others (14, 57, 62, 89).

**Table 2:** Current existing definitions of Dietary Reference Intake recommendations^a^

Recommendation	Definition
Acceptable Macronutrient Distribution Range (AMDR)	The percentage of total energy intake, expressed as a percentage of total energy, that is associated with a reduced risk of chronic disease and can provide adequate amounts of essential nutrients; intakes that fall above or below the range increase the potential for elevated risk of chronic disease or risk of inadequate consumption of essential nutrients
Estimated Energy Requirement (EER)	The average dietary energy intake that is predicted to maintain energy balance for a defined age, sex, weight, height, and level of physical activity, consistent with maintaining health
Estimated Average Requirement (EAR)	The average daily nutrient intake that is estimated to meet the requirement of half the healthy individuals in a particular life stage and gender group
Recommended Dietary Allowance (RDA)	The average daily dietary nutrient intake that is sufficient to meet the nutrient requirements of nearly all (97% to 98%) healthy individuals in a particular life stage and gender group
Adequate Intake (AI)	The recommended average daily intake level based on observed or experimentally determined approximations or estimates of nutrient intake by a group (or groups) of apparently healthy people that are assumed to be adequate; an AI is used when an EAR/RDA cannot be determined
Tolerable Upper Intake Level (UL)	The highest average daily nutrient intake level that is likely to pose no risk of adverse health effects to almost all individuals in the general population; as intake increases above the UL, the risk of adverse effects may increase
Chronic Disease Risk Reduction (CDRR)	The lowest level of intake for which there is sufficient strength of evidence to characterize a chronic disease risk reduction

a These definitions represent those of the Food and Nutrition Board of the National Academy of Medicine (38).
